# Underestimation of Pearson’s product moment correlation statistic

**DOI:** 10.1007/s00442-018-4233-0

**Published:** 2018-07-30

**Authors:** Rosalind K. Humphreys, Marie-Therese Puth, Markus Neuhäuser, Graeme D. Ruxton

**Affiliations:** 1 School of Biology, Dyer’s Brae House, University of St Andrews, St Andrews, KY16 9TH UK; 2 Institut für Medizinische Biometrie, Informatik und Epidemiologie (IMBIE), Universitätsklinikum Bonn (UKB), 53127 Bonn, Germany; 3 Department of Mathematics and Technology, RheinAhrCampus, Koblenz University of Applied Sciences, Remagen, Germany

**Keywords:** Association, Bias, Correlation, Pearson’s *r*, Sampling

## Abstract

Pearson’s product moment correlation coefficient (more commonly Pearson’s *r*) tends to underestimate correlations that exist in the underlying population. This phenomenon is generally unappreciated in studies of ecology, although a range of corrections are suggested in the statistical literature. The use of Pearson’s *r* as the classical measure for correlation is widespread in ecology, where manipulative experiments are impractical across the large spatial scales concerned; it is therefore vital that ecologists are able to use this correlation measure as effectively as possible. Here, our literature review suggests that corrections for the issue of underestimation in Pearson’s *r* should not be adopted if either the data deviate from bivariate normality or sample size is greater than around 30. Through our simulations, we then aim to offer advice to researchers in ecology on situations where both distributions can be described as normal, but sample sizes are lower than around 30. We found that none of the methods currently offered in the literature to correct the underestimation bias offer consistently reliable performance, and so we do not recommend that they be implemented when making inferences about the behaviour of a population from a sample. We also suggest that, when considering the importance of the bias towards underestimation in Pearson’s product moment correlation coefficient for biological conclusions, the likely extent of the bias should be discussed. Unless sample size is very small, the issue of sample bias is unlikely to call for substantial modification of study conclusions.

**Electronic supplementary material:**

The online version of this article (10.1007/s00442-018-4233-0) contains supplementary material, which is available to authorized users.

## Introduction

The essence of much of the statistical treatment of data is making inferences about an underlying population from a sample. For example, to explore the foraging behaviour of bumblebees we might collect a sample of 25 *Bombus terrestris* and explore the relationship between distance from the nest and body masses of these 25 individuals. We might expect that heavier individuals forage more widely. A natural way to quantify such a relationship would be through the Pearson’s product moment correlation coefficient (hereafter called Pearson’s *r*). Advice on the effective use of this statistical measure was recently summarised by Puth et al. (2014), who also presented the results of a survey of published papers that suggested that this measure of association was commonly used across biology. We found 26 papers published in Oecologia in the last 12 months, for which a primary outcome of the study involved calculation of this statistic (see Supplementary Information). In this hypothetical bumblebee example, interest lies not in the association between foraging range and body mass in this sample of 25 individuals, but in the underlying population. That is, we want to use the sample to make inferences about the association between these two traits in the underlying population of all individuals of this species that could theoretically have been included in this sample. In fact, Pearson’s *r* is unusual among commonly used statistical measures in that the sample measure is not an unbiased estimator of the population value. Specifically, the correlation measured on the sample tends to underestimate the correlation that exists in the whole population. This phenomenon is well known in the statistics literature (see below), but is generally not mentioned in statistics texts aimed at biologists. Consequently, this effect generally goes unacknowledged and unappreciated in the biology literature [but see brief mention on p. 566 of Sokal and Rohlf (1981), and more full treatment in DeGhett (2014) for exceptions]. The large spatial scale at which ecologists work makes manipulative experiments often impractical, so correlative studies are more common than in fields such as animal behaviour. For this reason, it is vital that ecologists use the classical measure of correlation (Pearson’s *r*) as effectively as they can. Our aim here is to provide a summary of existing evidence supplemented by our own investigations to offer researchers in ecology clear advice on what to do about the bias in Pearson’s *r*.

## Materials and methods

### Review of the existing literature

A range of correction factors are available in the statistics literature, which might be applied to the value of *r* calculated from a sample to reduce the bias, i.e., to make it more reflective of the population value. Shieh (2010) compared five such measures and found that the most effective of them was due to Olkin and Pratt (1958). Under this correction (generally called OPA after the original authors), if the sample measure is *r*, then they recommend correcting this to OPA(*r*) where

1 $$ {\text{OPA}}(r) = r\left( {1 + \frac{{1 - r^{2} }}{{2\left( {N - 4} \right)}}} \right). $$

Here, *N* is the sample size. However, Shieh points out that while such corrections can reduce the bias in estimation, they can increase the mean square error (MSE). That is, the corrected version is less likely to be consistently lower than the population value, but will on average be further away from the population value (reducing bias at a cost of reduced precision). Shieh further argued that the problem of increasing MSE was particularly acute for less strong correlations. Shieh offered the rule of thumb that if the magnitude of the sample *r* is less than 0.6, then no correction should be applied because the issue of increased uncertainty would dominate the issue of bias, but if the magnitude of *r* is greater than 0.6 then the OPA correction should be considered. If the sample size is very small (ten or less), then Sinsomboonthong et al. (2013) offer a method of correction based on jackknife sampling that might be more effective than OPA, but any improved performance would be relatively modest compared to the considerable increase in calculation complexity. Gorsuch and Lehmann (2010), on the basis of their simulations and a review of the literature, offer the rule of thumb that bias is strongest for moderate levels of *r* (with magnitudes between 0.3 and 0.7), but when *N* > 30 then issues of underestimation can be considered trivial. Zimmerman et al. (2003) also recommended the OPA correction after comparing it to alternatives in a simulation study (although note that they, in common with some other authors, utilise a formula with a “3” rather than “4” in the denominator). Although Pearson’s *r* is generally quite robust to deviation of the underlying assumption of normality in the underlying traits (Bishara and Hittner 2012), the corrections designed to reduce bias in bivariate normal data (like OPA) increase bias when underlying populations are non-normal (Bishara and Hittner 2015).

Thus, on the basis of previous literature, it is already possible to offer clear advice to the researcher in many situations. Correction for the issue of underestimation should not be adopted if either or both of the underlying distributions deviate from normality—in such a situation the issue of violation of the assumption of normality is more of a concern than that of underestimation, alternative measures of association may be appropriate; and Bishara and Hittner (2012) and Puth et al. (2014) provide clear advice on how to deal with this. Secondly, if sample size is greater than around 30, then the issue of underestimation is trivial, and so there is no benefit in complicating the analysis of data by applying a correction. In the next section, we focus on closing the gap in the literature, to offer advice on correction for the situation where both distributions are well approximated by the normal distribution and the sample size is low (*N* < 30). In our survey of 26 recent Oecologia papers, sample size was 30 or less in 6 cases and could not be determined from the paper in 12.

### Plan of our simulation studies

We evaluate the performance of different statistical approaches over 1000 samples drawn from a population with normal marginal distributions and a specified correlation (*ρ*), using the same methodology as Puth et al. (2014). We first consider the estimation of the 95% confidence interval for the population value of Pearson’s *r*. Puth et al. (2014) considered three methods for calculating the confidence interval: the BCa method of bootstrapping, the method due to both Muddapur (1988) and Jeyaratnam (1992) utilising *F* statistics, and the most commonly used version (due to Fisher, 1925) based on a z-statistic. For the first two of these, we compared the uncorrected versions used by Puth et al. (2014) with modifications where OPA correction is applied to all calculated *r* values. For the *z*-method, we compare the uncorrected method used in Puth et al. (2014) with one where after the value of *z* is calculated, it is then replaced by a value (z*) that was designed to correct for bias that causes* z* to be slightly larger than it should be. This correction is originally due to Hotelling (1953), was recommended by DeGhett (2014) and is given by:

2 $$ z^{*} = \left\{ {\begin{array}{*{20}ll} {z - \frac{3z + r}{4(N - 1)},\quad  {\text{if}}\, N > 10  } \\ {z - \frac{3z + r}{4(N - 1)} - \frac{{23z + 33r - 5r^{3} }}{{96(N - 1)^{2} }}, \quad {\text{if}}\, N \le 10} \\ \end{array} } \right. . $$

In Table 1, we evaluate this technique for samples drawn using the method described by Puth et al. (2014) with sample sizes *N* = 10, 20 and 30 for population correlations *ρ* = 0, 0.1, 0.3, 0.5, 0.7, 0.9. For each of the six methods, we calculate the mean coverage of the confidence intervals, defined as the fraction of 1000 confidence intervals that include the actual population value *ρ*. Values higher than 0.95 suggest that the confidence interval is too large, and values lower than 0.95 suggest that it is too narrow. For each combination of sample size and underlying correlation, we present a 3 × 2 set of numbers. For each of the three methods, we embolden whichever of the corrected or uncorrected situations offers coverage closer to 0.95, and we underline whichever of the six values is closest to 0.95.

**Table 1:** Estimations of the 95% confidence interval for the population value of Pearson’s *r* using three methods: BCa bootstrapping, *F* statistics and *Z*-statistics

*N*	Method	*ρ* = 0.0		*ρ* = 0.1		*ρ* = 0.3		*ρ* = 0.5		*ρ* = 0.7		*ρ* = 0.9	
		*r*	r*	*r*	r*	*r*	r*	*r*	r*	*r*	r*	*r*	r*
10	BCa	**0.954**	0.940	**0.958**	**0.942**	**0.955**	0.937	**0.946**	0.959	0.932	0.932	0.920	**0.925**
	*F*	**0.965**	0.976	**0.959**	0.970	**0.964**	0.965	**0.955**	0.980	**0.949**	0.968	**0.95**	0.957
	Fisher Z	0.964	**0.962**	**0.959**	0.975	**0.963**	0.968	**0.952**	0.965	**0.948**	0.973	**0.949**	0.957
20	BCa	**0.933**	0.929	0.921	**0.933**	0.923	**0.926**	**0.933**	0.914	**0.923**	0.912	**0.919**	0.899
	*F*	**0.953**	0.976	**0.932**	0.977	**0.951**	0.973	**0.947**	0.965	0.943	**0.951**	**0.944**	0.928
	Fisher Z	**0.952**	0.959	0.932	**0.955**	**0.95**	0.962	**0.944**	0.961	0.943	**0.956**	**0.943**	0.958
30	BCa	**0.931**	0.929	**0.939**	0.937	**0.949**	0.912	**0.928**	0.915	**0.940**	0.881	**0.937**	0.875
	*F*	**0.943**	0.980	**0.954**	0.985	**0.962**	0.970	**0.956**	0.973	**0.950**	0.939	**0.953**	0.903
	Fisher Z	**0.943**	0.963	**0.954**	0.965	0.962	**0.949**	**0.956**	0.964	**0.950**	0.953	**0.952**	0.946

The uncorrected BCa and *F*-methods were compared with OPA-corrected *r* values (labelled as r*). The uncorrected *Z*-method was compared with a version where the calculated value of *z* was replaced by a corrected value (z*). The corrections are evaluated with sample sizes *N* = 10, 20 and 30 for population correlations *ρ *= 0, 0.1, 0.3, 0.5, 0.7, 0.9. For each, the mean coverage of the confidence intervals is the fraction of 1000 confidence intervals that include the actual population value *ρ*. For each of the three methods, at each sample size and *ρ*, whichever of the corrected or uncorrected situations offers coverage closer to 0.95 is in bold. Whichever of the six values in the 3 × 2 grids considering all methods is closest to 0.95, with a given sample size and *ρ*, is bold and underlined

We then turn to testing the null hypothesis *ρ* = 0 (at the significance level *α* = 0.05) in Table 2 for the same combination of sample sizes and underlying *ρ* values. For *ρ *= 0 we give the type I error rate, otherwise we give the power. Again, there is a 3 × 2 combination of numbers in each cell, the first column being uncorrected and the second corrected. The three rows again refer to three methods considered in Puth et al. (2014). Firstly, we consider the standard method where t* is given by:

3 $$ t^{*} = \frac{r}{{\sqrt {\frac{{1 - r^{2} }}{N - 2}} }}. $$

t* is compared to a *t*-distribution with *N *− 2 degrees of freedom, the null hypothesis being rejected if the absolute value of t* is greater than the (1 – *α*/2) quantile of the respective *t*-distribution. Secondly, we consider a permutation test, where the null hypothesis is rejected if the observed value of *r* lies outside the 2.5 and 97.5 percentiles of a distribution of *r* scores calculated from permutations of the original sample. Finally, we use Fisher’s method, first calculating a *z* score as:

4 $$ z = 0.5\log_{\text{e}} \left( {\frac{1 + r}{1 - r}} \right). $$

**Table 2:** Testing the null hypothesis *ρ* = 0 (at the significance level *α* = 0.05) for *N* = 10, 20 and 30 for population correlations *ρ *= 0, 0.1, 0.3, 0.5, 0.7, 0.9

*N*	Method	*ρ* = 0.0		*ρ* = 0.1		*ρ* = 0.3		*ρ* = 0.5		*ρ* = 0.7		*ρ* = 0.9	
		*r*	r*	*r*	r*	*r*	r*	*r*	r*	*r*	r*	*r*	r*
10	t*	0.023	**0.032**	0.039	**0.069**	0.116	**0.181**	0.323	**0.361**	0.665	**0.738**	0.978	**0.994**
	Permutation	0.059	**0.046**	**0.061**	0.056	0.123	**0.158**	0.315	**0.321**	0.652	**0.678**	**0.982**	0.979
	Fisher* Z*	**0.032**	0.013	**0.047**	0.026	**0.126**	0.080	**0.339**	0.236	**0.692**	0.582	**0.99**	0.974
20	t*	0.019	**0.031**	0.061	**0.075**	0.242	**0.281**	0.652	**0.662**	0.952	**0.957**	**1**	**1**
	Permutation	0.045	**0.049**	0.066	**0.094**	**0.264**	0.247	**0.646**	0.642	0.96	**0.962**	**1**	**1**
	Fisher* Z*	**0.027**	0.024	**0.061**	0.053	**0.251**	0.238	**0.631**	0.587	**0.969**	0.938	**1**	**1**
30	t*	**0.030**	0.025	**0.072**	0.069	0.365	**0.397**	**0.828**	0.809	0.994	**0.995**	**1**	**1**
	Permutation	**0.053**	0.041	0.072	**0.084**	**0.375**	0.372	0.827	**0.838**	**0.993**	0.991	**1**	**1**
	Fisher* Z*	**0.030**	0.017	0.068	**0.072**	**0.387**	0.333	**0.836**	0.813	0.991	**0.993**	**1**	**1**

For *ρ *= 0 the type I error rate is given, otherwise power is given. For each *ρ*, uncorrected values are given in the first column (*r*) and corrected (r*) in the second. The three methods considered were: t*, a permutation test and Fisher’s *Z* method. To implement correction for underestimation, the t* and permutation methods had their calculated values of *r* replaced by the OPA-corrected value. For Fisher *Z*, *z* values were replaced by the appropriate corrected value z*. For each pair of uncorrected or corrected values, whichever offers the highest power (or type I error rate closest to the nominal 0.05 level) is in bold. For each group of six values whichever method performs best of the six (in terms of highest power or type I error rate) is bold and underlined

Then we compare

5 $$ Z = \frac{z}{{\sqrt {\frac{1}{N - 3}} }}, $$

with the (1 − *α*/2) quantile of the standard normal distribution (i.e., 1.96 if *α* = 0.05), rejecting the null hypothesis if the absolute value of the calculated value is bigger than or equal to 1.96. To implement correction for underestimation, for the first two methods we replace all calculated values of *r* with the OPA-corrected value at all stages of the procedure; for the final method, we replace *z* with the appropriate corrected value z* as defined above. Results are shown in Table 2; for each combination of sample size and the three methods, we calculate the power (or type I error rate for *ρ *= 0), using both the uncorrected and corrected methods (columns “*r*” and “r*”, respectively). For each pair of uncorrected or corrected values, we embolden whichever offers the higher power (or type I error rate closest to the nominal 0.05 level). For each group of six values, we underline whichever uncorrected or corrected method performs best of the six (in terms of highest power or type I error rate closest to the nominal level).

**Fig. 1:**
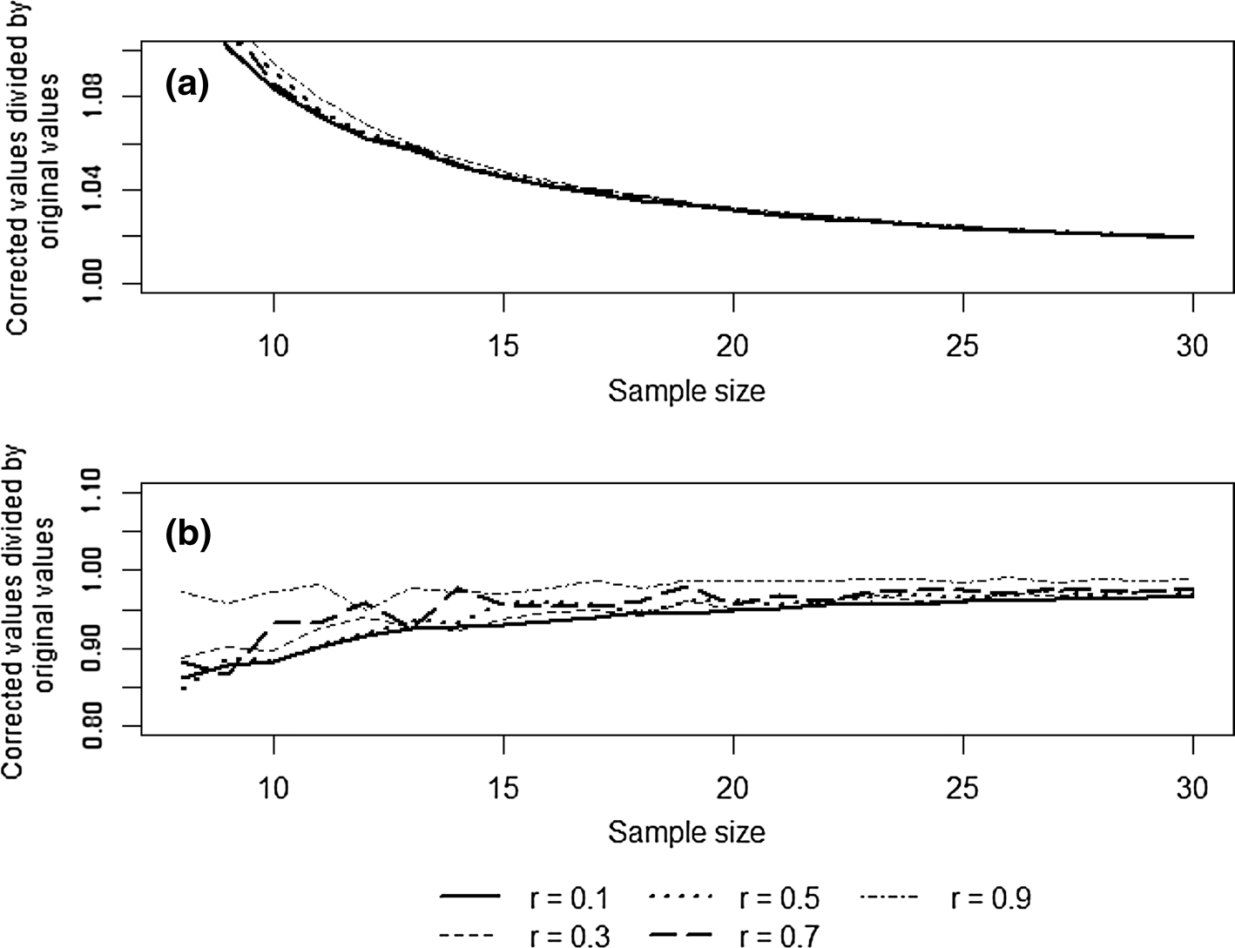
**a** OPA-corrected values divided by the original *r* value, for sample sizes 8–30 and *r* values 0.1, 0.3, 0.5, 0.7 and 0.9. **b** Corrected *r* values divided by the original *r* values produced via the *z*-method using Eq. (2), for sample sizes 8–30 and *r* values 0.1, 0.3, 0.5, 0.7 and 0.9. The corrected *r* values are recovered after the correction has been made to Fisher’s *z* by the formula given in Eq. (6)

In Fig. 1a, we then plot the OPA-corrected value divided by the original *r* value calculated from a sample, for sample sizes 8–30 and *r* values 0.1, 0.3, 0.5, 0.7 and 0.9. In Fig. 1b, we do the same for *z* correction where, after the correction has been made to Fisher’s *z*, the corrected *r* value is recovered using:

6 $$ r = \frac{{\exp \left( {2z^{*} } \right) - 1}}{{\exp \left( {2z^{*} } \right) + 1}}. $$

**Fig. 2:**
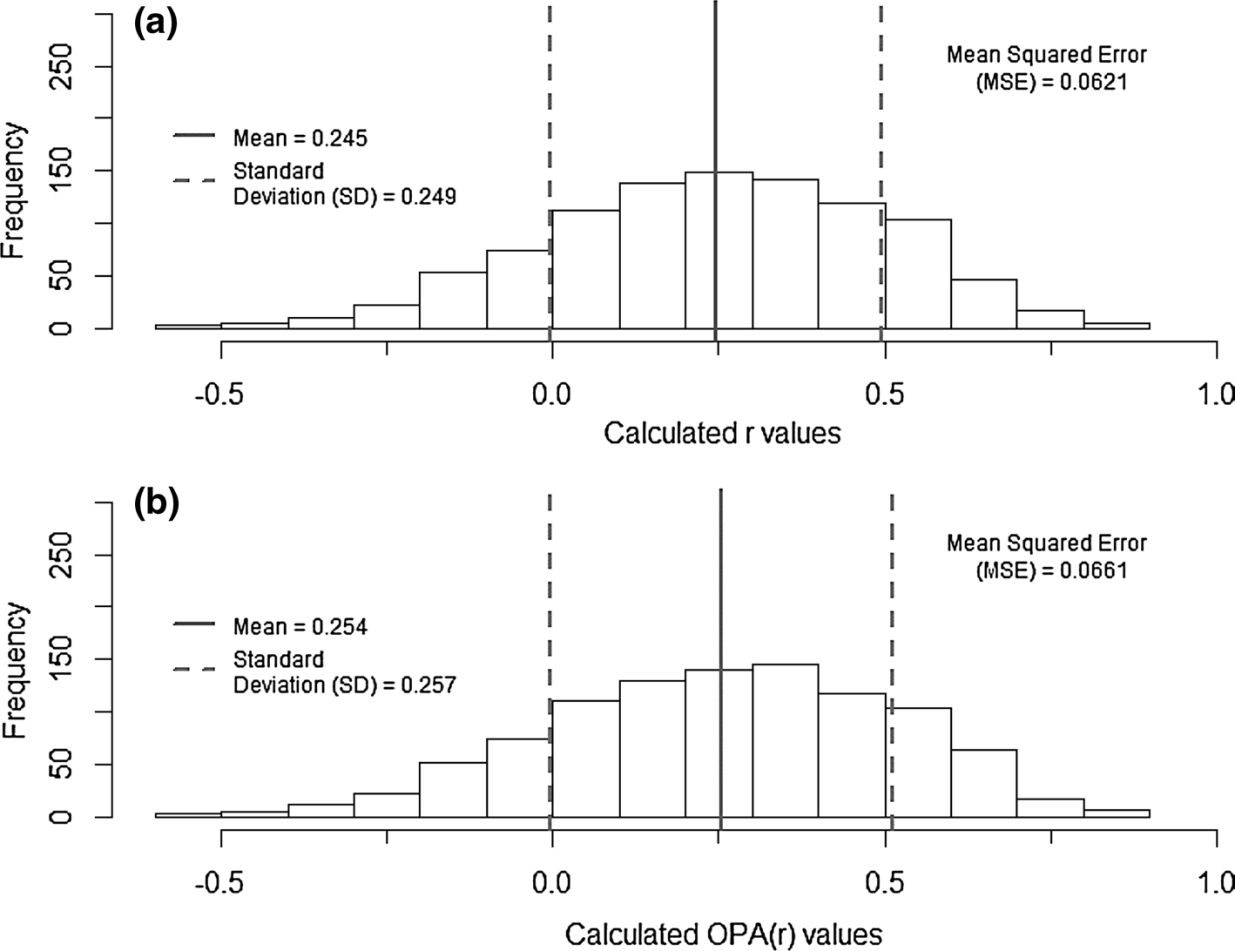
**a** Histogram of *r* values calculated from 1000 samples with *N *= 15 and *ρ* = 0.25; mean (full line), standard deviation (SD) (dashed line) and mean squared error (MSE) are shown. **b** Histogram of OPA(*r*) values calculated from 1000 samples with *N* = 15 and *ρ* = 0.25; mean (full line), standard deviation (SD) (dashed line) and mean squared error (MSE) are shown

Finally, in Fig. 2a, we investigate the spread of sample values by plotting the frequency of *r* values calculated from 1000 samples with *N *= 15 and *ρ* = 0.25, drawing attention to the mean, standard deviation and mean squared error. In Fig. 2b, we show the same for the OPA(*r*)-corrected values for the same sample size and *r*.

## Results

Table 1 gives no evidence to support adoption of the OPA correction for calculation of confidence intervals. Regardless of the method used, correction does not cause a general tendency to give coverage values closer to the nominal 0.95 value. There is perhaps a tendency for correction to lead to confidence intervals that are too wide (hence with coverage above 0.95), but this tendency is not consistent.

We now turn to Table 2 for testing the null hypothesis that *ρ *= 0. Considering type I error rate first, we find that all methods are overwhelmingly conservative, with type I error rates being mostly below 0.05: something that correction does not substantially change. Turning to power (with *ρ *= 0.1, 0.3, 0.5, 0.7, 0.9), we find unsurprisingly that the power for all (corrected and uncorrected) methods increases with sample size and with the population value of *ρ *. Puth et al. (2014) did not find a strong difference in power between the three uncorrected methods, and our results agree with this. We find the same to be true when comparing powers of the three corrected versions. Most importantly, for any specific method we do not observe correction offering a conspicuous and consistent improvement in power. Hence, we do not find strong evidence in support of correcting calculated correlation coefficients as part of null hypothesis testing.

Figure 1 shows that it appears that—irrespective of the size of *r*—where sample sizes are > 15, there is very little difference between *r* and OPA(*r*), a similar trend can be seen for the correction to *z* in Fig. 1b. From Fig. 2, it can then be observed that, firstly, such small samples can produce a broad range of different *r* values across our 1000 samples. Secondly, the mean *r* of the 1000 samples is lower than the population value of 0.25 (i.e. it is downwardly biased, as expected), but the mean value of OPA(*r*) is noticeably (slightly) closer to 0.25 (so the correction slightly reduced bias on average). Finally, the standard deviation and the mean squared error of the OPA-corrected values are larger than for the *r* values; this suggests that the reduction in bias through the use of OPA corrections comes at a cost in imprecision—and imprecision is a more dominant feature than bias in this example situation.

## Discussion

On the basis of our survey of the literature and our own simulations, we can offer clear advice to the many researchers in ecology who use Pearson’s *r* in the statistical treatment of their data.

Firstly, they should be aware that the value measured on their sample will be more often biased towards underestimating than overestimating the true value of the underlying population they are interested in. This possible bias was not discussed in any of the papers in our survey.

Further, they should be aware that testing the null hypothesis of no association is conservative, rejecting the null hypothesis when it is true at lower than the nominal rate* α*. This hypothesis was tested in 21 of the 26 papers in our survey; but none of these discussed the conservatism of this test.

Next, they should not attempt any of the methods offered in the literature for correcting bias. No method yet developed offers consistently reliable performance. Additionally, the fact that the standard deviation of OPA-corrected values (Fig. 2b) was greater than that for the *r* values (Fig. 2a) illustrates that any reduction in bias through corrections could increase imprecision.

Finally, when discussing the importance of this bias towards underestimation for the biological conclusions to be drawn from their study, they should quantify the likely extent of this bias. We see in Fig. 1a that (regardless of the size of the actual correlation *ρ*) as long as *N *> 15, the difference between *r* and OPA(*r*) is less than 5% of *r*. Sample size was less than 15 in 3 papers out of 26 in our survey. Thus, unless sample size is very small, the issue of sample bias is unlikely to call for substantial modification of biological conclusions. For such sample sizes, statistical power is likely to be very low (see Tables 1, 2) and thus imprecision may often be a greater concern than bias even in this situation. In our survey of 26 papers, 1 provided a confidence interval, and none of the others discussed precision in any way. We have demonstrated here three simple and general ways that such a confidence interval can be calculated as a very useful aid to discussing imprecision of estimation.

## Electronic supplementary material

Below is the link to the electronic supplementary material.

Supplementary material 1 (PDF 143 kb)
