# Supplementary material for: Underestimation of Pearson’s product moment correlation statistic
Source: Oecologia. 2018 Jul 30;189(1):1–7. doi: 10.1007/s00442-018-4233-0 (PMC6323088; doi:10.1007/s00442-018-4233-0)
Supplement: Supplementary file 1 — Supplementary material 1 (PDF 143 kb) [file 442_2018_4233_MOESM1_ESM.pdf]

Supplementary Information

Reference	Calculated r value*	Correct for underestimation?	Sample size? ^	Bias discussed?	Test if the value is significantly different from 0?	Supply a confidence interval?
Mausolf et al. (2018)	-0.66	No	?	No	Yes	No
Krashevska et al. (2018)	0.44	No	?	No	Yes	No
Atkinson et al. (2018)	0.64	No	55	No	Yes	Yes
Frances and McCauley (2018)	-0.99	No	5	No	Yes	No
Zhou et al. (2018)	0.41	No	320	No	Yes	No
Chauvin et al. (2018)	0.59	No	79	No	Yes	No
Meller et al. (2018)	0.88	No	?	No	No	No
Atuo and O'Connell (2018)	-0.02	No	?	No	No	No
Gabor et al. (2018)	-0.07	No	11	No	Yes	No
Chisté et al. (2018)	0.35	No	140	No	Yes	No
Spiesman et al. (2018)	0.91	No	21	No	No	No
Ochoa-López et al. (2018)	-0.57	No	?	No	Yes	No
Calf et al. (2018)	0.07	No	?	No	Yes	No
Keller et al. (2018)	0.58	No	?	No	Yes	No
Quesnel et al. (2018)	0.06	No	247	No	Yes	No
Chen et al. (2017)	-0.33	No	?	No	Yes	No
Castle et al. (2017)	-0.55	No	?	No	Yes	No
Wilkins et al. (2017)	0.39	No	35	No	Yes	No
Buler et al. (2017)	0.95	No	15	No	Yes	No
Zukswert and Prescott (2017)	0.69	No	12	No	Yes	No
Bajcz and Drummond (2017)	0.713	No	?	No	No	No
Osada and Hiura (2017)	0.17	No	?	No	Yes	No
Cadahía et al. (2017)	0.56	No	?	No	Yes	No
Saino et al. (2017)	-0.131	No	52	No	Yes	No
Rosa and Saastamoinen (2017)	0.8	No	124	No	No	No
Reichert et al. (2017)	-0.008	No	30	No	Yes	No

*first encountered value in the results section if there were multiple Pearson's product moment correlation tests
^ recorded as '?' where sample size could not be determined

Supplementary Information

References:

Atkinson CL et al. (2018) Determinants of food resource assimilation by stream insects along a tropical elevation gradient. Oecologia. doi: 10.1007/s00442-018-4142-2

Atuo FA, O'Connell TJ (2018) Superpredator proximity and landscape characteristics alters nest site selection and breeding success of a subordinate predator. Oecologia 186:817-829. doi: 10.1007/s00442-018-4071-0

Bajcz AW, Drummond FA (2017) Bearing fruit: flower removal reveals the trade-offs associated with high reproductive effort for lowbush blueberry. Oecologia 185:13-26. doi: 10.1007/s00442-017-3908-2

Buler JJ, Lyon RJ, Smolinsky JA, Zenzal TJ, Moore FR (2017) Body mass and wing shape explain variability in broad-scale bird species distributions of migratory passerines along an ecological barrier during stopover. Oecologia 185:205-212. doi: 10.1007/s00442-017-3936-y

Cadahía L et al. (2017) Advancement of spring arrival in a long-term study of a passerine bird: sex, age and environmental effects. Oecologia 184:917-929. doi: 10.1007/s00442-017-3922-4

Calf OW, Huber H, Peters JL, Weinhold A, van Dam NM (2018) Glycoalkaloid composition explains variation in slug resistance in Solanum dulcamara. Oecologia 187:495-506. doi: 10.1007/s00442-018-4064-z

Castle SC et al. (2017) Nutrient limitation of soil microbial activity during the earliest stages of ecosystem development. Oecologia 185:513-524. doi: 10.1007/s00442-017-3965-6

Chauvin KM, Asner GP, Martin RE, Kress WJ, Wright SJ, Field CB (2018) Decoupled dimensions of leaf economic and anti-herbivore defense strategies in a tropical canopy tree community. Oecologia 186:765-782. doi: 10.1007/s00442-017-4043-9

Chen H et al. (2017) Root chemistry and soil fauna, but not soil abiotic conditions explain the effects of plant diversity on root decomposition. Oecologia 185:499-511. doi: 10.1007/s00442-017-3962-9

Chisté MN, Mody K, Kunz G, Gunczy J, Blüthgen N (2018) Intensive land use drives small-scale homogenization of plant- and leafhopper communities and promotes generalists. Oecologia 186:529-540. doi: 10.1007/s00442-017-4031-0

Frances DN, McCauley SJ (2018) Warming drives higher rates of prey consumption and increases rates of intraguild predation. Oecologia. doi: 10.1007/s00442-018-4146-y

Gabor CR, Knutie SA, Roznik EA, Rohr JR (2018) Are the adverse effects of stressors on amphibians mediated by their effects on stress hormones? Oecologia 186:393-404. doi: 10.1007/s00442-017-4020-3

Keller KR, Carabajal S, Navarro F, Lau JA (2018) Effects of multiple mutualists on plants and their associated arthropod communities. Oecologia 186:185-194. doi: 10.1007/s00442-017-3984-3

Krashevska V et al. (2018) Micro-decomposer communities and decomposition processes in tropical lowlands as affected by land use and litter type. Oecologia 187:255-266. doi: 10.1007/s00442-018-4103-9

Mausolf K et al. (2018) Legacy effects of land-use modulate tree growth responses to climate extremes. Oecologia. doi: 10.1007/s00442-018-4156-9

Meller K, Piha M, Vähätalo AV, Lehikoinen A (2018) A positive relationship between spring temperature and productivity in 20 songbird species in the boreal zone. Oecologia 186:883-893. doi: 10.1007/s00442-017-4053-7

Ochoa-López S, Rebollo R, Barton KE, Fornoni J, Boege K (2018) Risk of herbivore attack and heritability of ontogenetic trajectories in plant defense. Oecologia 187:413-426. doi: 10.1007/s00442-018-4077-7

Supplementary Information

Osada N, Hiura T (2017) How is light interception efficiency related to shoot structure in tall canopy species? Oecologia 185:29-41. doi: 10.1007/s00442-017-3926-0

Quesnel L, King WJ, Coulson G, Festa-Bianchet M (2018) Tall young females get ahead: size-specific fecundity in wild kangaroos suggests a steep trade-off with growth. Oecologia 186:59-71. doi: 10.1007/s00442-017-4003-4

Reichert S et al. (2017) Telomere length measurement by qPCR in birds is affected by storage method of blood samples. Oecologia 184:341-350. doi: 10.1007/s00442-017-3887-3

Rosa E, Saastamoinen M (2017) Sex-dependent effects of larval food stress on adult performance under semi-natural conditions: only a matter of size? Oecologia 184:633-642. doi: 10.1007/s00442-017-3903-7

Saino N et al. (2017) Wing morphology, winter ecology, and fecundity selection: evidence for sex-dependence in barn swallows (Hirundo rustica). Oecologia 184:799-812. doi: 10.1007/s00442-017-3918-0

Spiesman BJ, Kummel H, Jackson RD (2018) Carbon storage potential increases with increasing ratio of C4 to C3 grass cover and soil productivity in restored tallgrass prairies. Oecologia 186:565-576. doi: 10.1007/s00442-017-4036-8

Wilkins LGE et al. (2017) Maternal allocation of carotenoids increases tolerance to bacterial infection in brown trout. Oecologia 185:351-363. doi: 10.1007/s00442-017-3952-y

Zhou Y, Boutton TW, Wu XB, Wright CL, Dion AL (2018) Rooting strategies in a subtropical savanna: a landscape-scale three-dimensional assessment. Oecologia 186:1127-1135. doi: 10.1007/s00442-018-4083-9

Zukswert JM, Prescott CE (2017) Relationships among leaf functional traits, litter traits, and mass loss during early phases of leaf litter decomposition in 12 woody plant species. Oecologia 185:305-316. doi: 10.1007/s00442-017-3951-z
